# Fusion of the Endoplasmic Reticulum and Mitochondrial Outer Membrane in Rats Brown Adipose Tissue: Activation of Thermogenesis by Ca^2+^

**DOI:** 10.1371/journal.pone.0009439

**Published:** 2010-03-02

**Authors:** Leopoldo de Meis, Luisa A. Ketzer, Rodrigo Madeiro da Costa, Ivone Rosa de Andrade, Marlene Benchimol

**Affiliations:** 1 Instituto de Bioquímica Médica, Centro de Ciências da Saúde, Universidade Federal do Rio de Janeiro, Rio de Janeiro, Brasil; 2 Laboratório de Ultraestrutura Celular, Universidade Santa Úrsula, Rio de Janeiro, Brasil; Universität Heidelberg, Germany

## Abstract

Brown adipose tissue (BAT) mitochondria thermogenesis is regulated by uncoupling protein 1 (UCP 1), GDP and fatty acids. In this report, we observed fusion of the endoplasmic reticulum (ER) membrane with the mitochondrial outer membrane of rats BAT. Ca^2+^-ATPase (SERCA 1) was identified by immunoelectron microscopy in both ER and mitochondria. This finding led us to test the Ca^2+^ effect in BAT mitochondria thermogenesis. We found that Ca^2+^ increased the rate of respiration and heat production measured with a microcalorimeter both in coupled and uncoupled mitochondria, but had no effect on the rate of ATP synthesis. The Ca^2+^ concentration needed for half-maximal activation varied between 0.08 and 0.11 µM. The activation of respiration was less pronounced than that of heat production. Heat production and ATP synthesis were inhibited by rotenone and KCN.

Liver mitochondria have no UCP1 and during respiration synthesize a large amount of ATP, produce little heat, GDP had no effect on mitochondria coupling, Ca^2+^ strongly inhibited ATP synthesis and had little or no effect on the small amount of heat released. These finding indicate that Ca^2+^ activation of thermogenesis may be a specific feature of BAT mitochondria not found in other mitochondria such as liver.

## Introduction

In some tissues, mitochondria are physically linked to the endo/sarcoplasmic reticulum (ER). This has been observed in liver cells, mouse embryonic fibroblasts, HeLa cells, melanocytes, skeletal muscle and cardiac myocyte [1]–[6]. This connection is referred to as mitochondria-associated ER membrane (MAM). Lipids and Ca^2+^ are exchanged between the two sub cellular compartments through MAM [4]. The mitochondrial Ca^2+^ concentration is regulated by MAM, allowing it to rise to a level adequate to enhance mitochondrial bioenergetics activity while simultaneously preventing a rise to a level that triggers apoptosis. Excellent reviews about MAM and its involvement in mitochondria Ca^2+^ regulation have been recently published [4], [6], [7].

Brown adipose tissue (BAT) is capable of rapidly converting fat stores to heat and has been used as a model system for the understanding of nonshivering heat production and mechanism of energy wasting to control obesity [8]–[10]. BAT is found in small rodents, newborn children and in adult's humans [11]–[15] Within BAT cells, the main source of heat production is the mitochondria. Two specific features of BAT mitochondria, which differentiate them from the mitochondria found in other tissues are (i) the presence of uncoupling protein isoform 1 (UCP1) which is specifically found in BAT [8]–[11] and (ii) the presence of a sarco/endoplasmic reticulum Ca^2+^ transport ATPase isoform 1 (SERCA 1) attached to the cristae of BAT mitochondria [16]. The isoform found in BAT is the same as that found in both BAT endoplasmic reticulum and in skeletal muscle sarcoplasmic reticulum [16]–[18]. As far as we know, up to now, SERCA has been identified only in BAT mitochondria.

BAT thermogenesis is activated by adrenergic stimulation, which promotes the raise of both cytosolic fatty acids and Ca^2+^ concentrations [8]–[10], [19], [20]. There seems to be more than one system contributing to the regulation of BAT mitochondrial thermogenesis [20]–[22] but the best known involves the mitochondrial uncoupling protein 1 (UCP 1), fatty acids and GDP. UCP 1 is a protein inserted in the mitochondrial inner membrane, which, in the presence of GDP is impermeable to H^+^. In this case, the mitochondria are coupled and the energy derived from respiration is used for ATP synthesis. After adrenergic stimulation, the rise of cytosolic fatty acids displaces GDP from UCP1 increasing its H^+^ permeability, thus uncoupling the mitochondria and dissipating the energy derived from respiration into heat [8]–[10], [20].

In a previous report, using isolated mitochondria, we found that the rise of Ca^2+^ concentration to a level similar to that observed in BAT cytosol during adrenergic stimulation promotes an increase in mitochondrial thermogenic activity [16]. In this report, we observed that, similar to skeletal muscle, BAT endoplasmic reticulum fuses with BAT mitochondria forming MAM. Immunolabeling with monoclonal anti-SERCA 1 antibodies and gold-labeled goat anti-mouse IgB suggest that SERCA 1 is transferred from the ER to BAT mitochondria through MAM.

## Results

### Electron Microscopy

**Figure 1:**
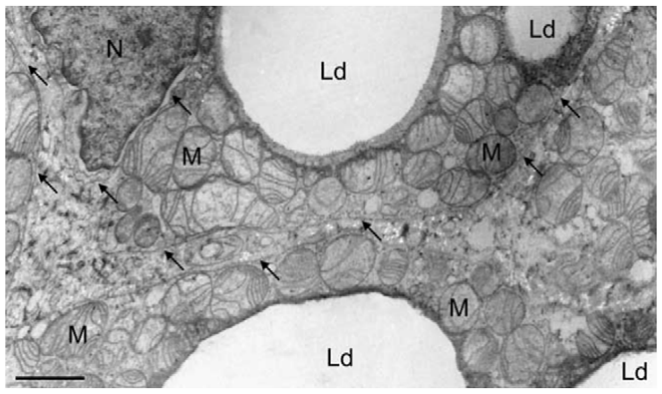
Electron microscopy of brown adipose tissue (BAT). Ultra-thin section of a tissue fragment showing the main constituents of a BAT cell. Large lipid droplets (Ld) are present, surrounded by mitochondria (M). The endoplasmic reticulum (arrows) can be seen all over the cytosol, but mainly close to mitochondria. N, nucleus. Bar = 1 µm.

**Figure 2:**
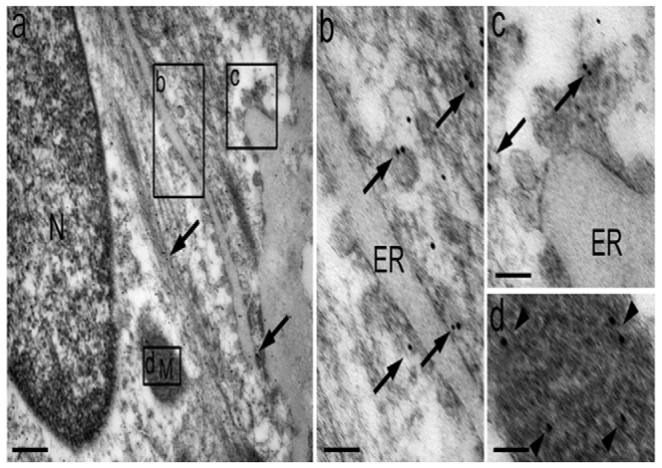
Immunolabeling of SERCA 1 using a gold-conjugated antibody. (a) BAT cell presenting a nucleus (N) surrounded by endoplasmic reticulum (arrows) and mitochondria (M) that are positively labeled for SERCA 1. (b and c) Note that protruding structures from the endoplasmic reticulum are also positive for SERCA 1. (d) A higher magnification of the mitochondria; note that it is positive for SERCA 1, mainly in the inner membrane. Bar: a, 250 nm, b and c, 50 nm, d, 30 nm.

**Figure 3:**
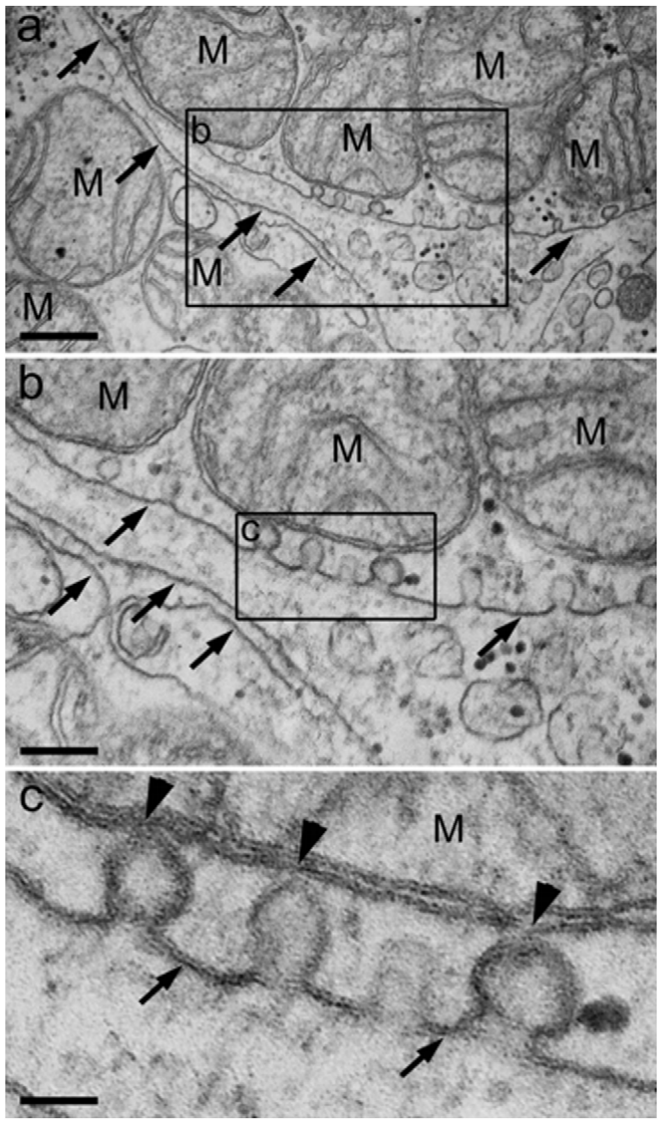
Mitochondria and ER contact. a) Ultrastructural views of a BAT cell showing a high number of mitochondria (M) and ER (arrows). (b) Budding-like structures are seen protruding from the ER (inset in a) and attached to mitochondria (arrowheads), as seen in a higher magnification in (c). Bars: a, 500 nm, b, 250 nm and c, 180 nm.

**Figure 4:**
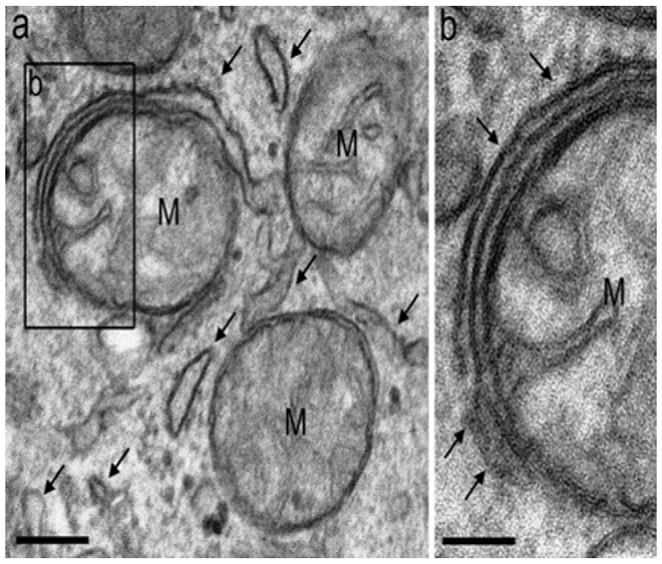
BAT electron micrograph. In (a), mitochondria (M) are in contact with the membranes of the endoplasmic reticulum (ER) (arrows). Notice that the ER wraps around the mitochondria, creating a trilaminar structure. (b) Higher magnification of the (a) inset showing a close view of the intimate proximity between mitochondria (M) and ER (arrows). Bars: a, 500 nm and b, 100 nm.

**Figure 5:**
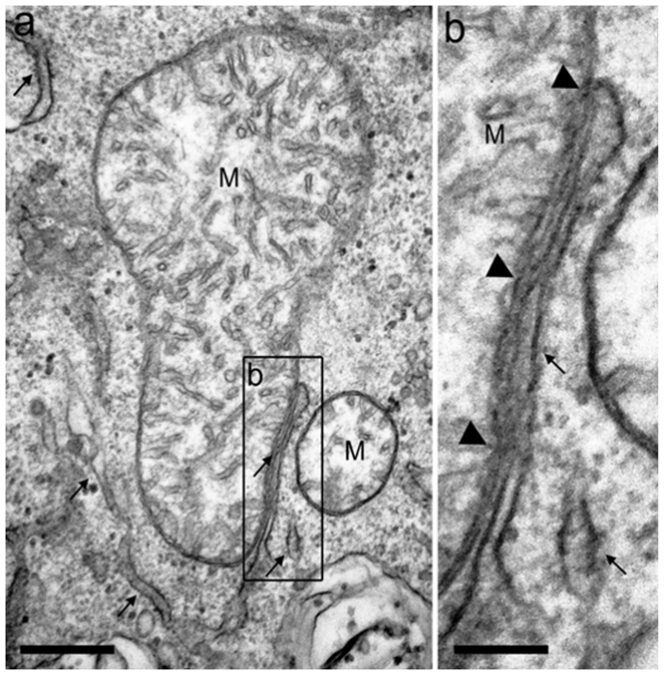
Mitochondria (M) attached to endoplasmic reticulum (a). Part (b), a higher magnification of the inset of panel (a), shows the contact site (arrow heads) between mitochondria (M) and an endoplasmic reticulum profile. Bars: a, 500 nm and b, 100 nm.

**Figure 6:**
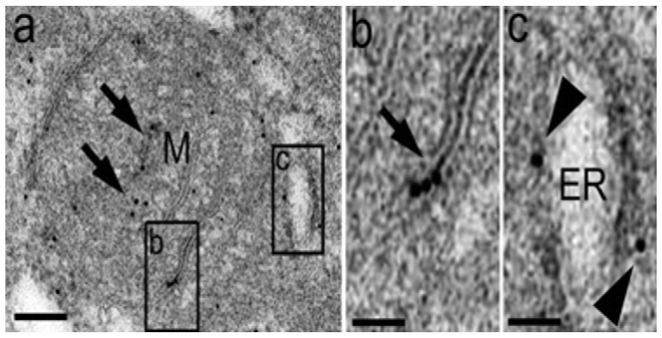
Immunolocalization of SERCA 1 in BAT mitochondria and ER. Notice that SERCA 1 immunolabeling is seen both in mitochondria (M, arrows) and on endoplasmic reticulum profiles (arrowheads). Higher magnification of SERCA 1 immunolabeling in the inner mitochondrial membrane (b, arrow) and endoplasmic reticulum (c, arrowheads). Bars: a, 250 nm, b and c, 85 nm.

**Figure 7:**
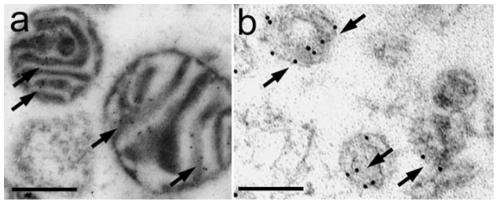
Electron microscopy of isolated mitochondria and microsomes immunolabeled with anti-SERCA 1 antibody. Positive labeling is observed on mitochondrial cristae (a, arrows) and in the microsomal membrane (b, arrows). Bars: a, 500 and b, 200 nm.

**Figure 8:**
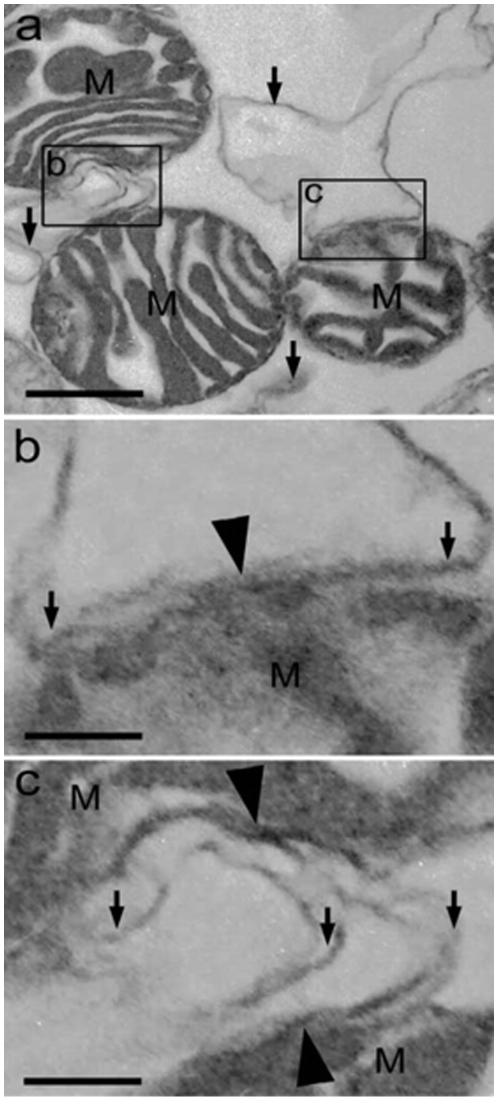
Isolated mitochondria. Notice ER fragments attached to mitochondria (arrows) even after differential centrifugation in a Percoll gradient. Bars: a, 500 nm, b and c, 125 nm.

BAT cells did contain a large number of mitochondria and an extended ER network that surrounded mitochondria, the nucleus and the cell lipid deposits (Fig. 1). The shape and diameter of the ER varied, ranging from straight neat tubules to large and convoluted structures. Protruding from the ER there were globular structures (Figs. 2 and 3). In the vicinity of mitochondria, these protrusions enter in contact with the outer mitochondrial membrane (Fig. 3). The images of Figs. 3, 4, 5 suggest that, after establishing contact, the ER projections propitiate the fusion of the ER membrane with the mitochondrial outer membrane. Immunolabeling with monoclonal anti-SERCA 1 antibodies and gold-labeled goat anti-mouse IgG revealed the presence of SERCA 1 in the ER, ER projections and in mitochondrial cristae (Figs. 2, 6 and 7a). These images raise the possibility that, in addition to lipids and Ca^2+^, SERCA 1 (Figs. 4 and 6) could also be transferred from the ER to mitochondria via MAM. Immunolabeling was clearly seen in isolated mitochondria and vesicles isolated from the ER by differential centrifugation (Fig. 7). In isolated mitochondria, we observed that some of them retain ER attached to the outer membrane (Fig. 8), indicating that the fusion between the two structures can be strong enough to resist tissue homogenization and centrifugation in a Percoll gradient.

### Thermogenesis

**Figure 9:**
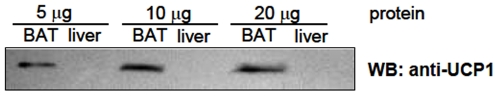
UCP 1 expression in BAT and liver mitochondria. Five to twenty micrograms of mitochondria derived from BAT and liver were used to load the gel. The immunodetection was obtained with UCP 1-specific polyclonal antibody.

**Figure 10:**
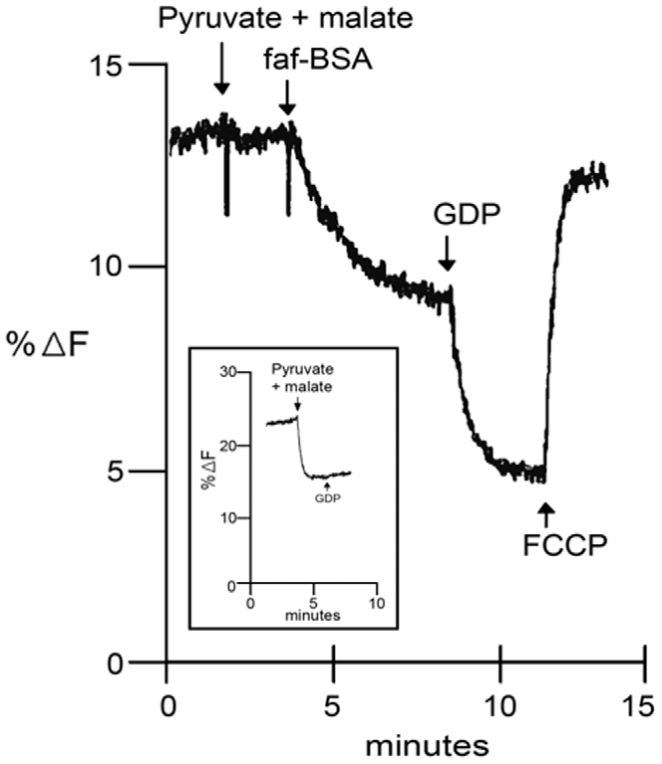
The main frame shows the membrane electrochemical potential (Δψ) formation of BAT mitochondria after successive additions of faf-BSA and GDP, and Δψ collapse by the proton ionophore FCCP. The inset shows Δψ formation of liver mitochondria and the lack of effect of 1 mM GDP.

The mechanism by which Ca^2+^ released in the cell during adrenergic stimulation activates BAT thermogenesis is not yet clear. The finding of SERCA 1 in BAT mitochondria (Fig. 7) raises the possibility that the activation by Ca^2+^ is somehow related to the mitochondrial SERCA 1. Therefore, in the following experiments, we tested the effects of GDP, Ca^2+^, and lipids in BAT isolated mitochondria. As a control, some of the experiments performed with BAT mitochondria were repeated with liver mitochondria which has UCP2 [23], [24] but does not contain UCP 1 (Fig. 9). The aim was to verify if the effects observed with BAT were specific of this tissue or if they could also be observed in other tissues containing different UCP isoforms such as liver mitochondria. Initially we measured the effects of GDP and Ca^2+^, in the formation of an electrochemical membrane potential (ΔΨ). BAT mitochondria were not able to form a Δψ after the single addition of the respiratory substrates pyruvate and malate [Fig. 10). Removal of lipids with excess fatty free serum albumin (faf-BSA) promoted the formation of a ΔΨ which was further enhanced by GDP. The same profile was observed if GDP was added before faf-BSA (data not shown). BAT ΔΨ formation was not altered by Ca^2+^ concentrations varying from 0.1 up to 2.0 µM, (data not shown). Different from BAT, in liver mitochondria a ΔΨ was formed after the addition of respiratory substrate without the need of adding either faf-BSA or GDP (Fig 10 inset). In both BAT and in liver mitochondria, the ΔΨ formed was collapsed by the proton ionophore FCCP. In conclusion, GDP promote a significant ΔΨ increase in BAT but had no measurable effect in liver mitochondria.

### Respiration, Heat Production and ATP Synthesis

**Figure 11:**
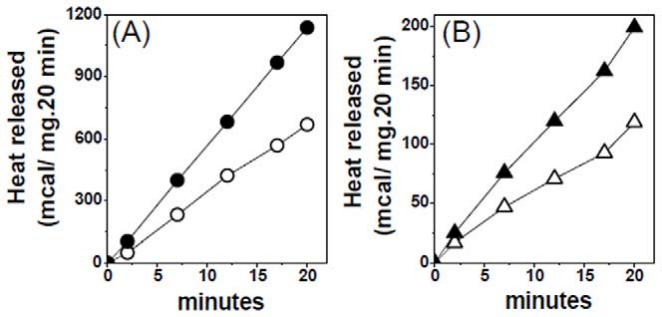
Effect of Ca^2+^ on (A) the rate of uncoupled and (B) coupled mitochondria. The figure shows a typical experiment. Open symbols without Ca^2+^ (1mM EGTA) and closed symbols 2µM Ca^2+^ (0.1 mM EGTA and 0.1 mM CaCl_2_).

After the single addition of respiratory substrates pyruvate and malate BAT mitochondria were uncoupled and in this condition there was practically no ATP synthesized (Table 1) but both the rates of respiration and the calorimetrically measured heat production were fast. We now show that the addition of a low Ca^2+^ concentration (∼2 µM calculated), promoted a significant increase of the three parameters measured, i.e. calorimetric heat production, respiration and ATP synthesis. The small amount of ATP synthesized in presence of Ca^2+^ was not inhibited by oligomycin. Fig. 11 (A, uncoupled; B, coupled) shows a typical experiment on the Ca^2+^ effect on the rate of heat production and Table 1 the average ± SE of different experiments of both respiration and heat production. An intriguing finding in Table 1 was that the increment of oxygen consumption promoted by Ca^2+^ (28%) was less pronounced than the increment of heat production (60%). In three experiments, the Ca^2+^ concentration needed for half-maximal activation of heat production was found to vary between 0.08 and 0.11 µM.

**Table 1:** Heat measured, oxygen consumed and ATP synthesized by BAT mitochondria.

BAT mitochondria	Heat released mcal/mg.20 min		Respiration µmol ½ O_2_/mg . 20 min		ATP synthesis µmol/mg . 20 min	
	EGTA	Ca^2+^	EGTA	Ca^2+^	EGTA	Ca^2+^
Uncoupled	627**^a^**±48 (28)	1,005**^a^**±64 (24)	9.9^c^±0.9(12)	12.7^c^±1 (12)	0.10 **^e^**±0.05 (15)	0.42**^e^**±0.10 (15)
Coupled	146**^b^**±5(18)	225**^b^**±19 (18)	2.4 **^d^**±0,2 (7)	2.9**^d^**±0,2 (7)	2.1**^f^**±0.3 (16)	2.5 **^f^**±0.3(16)

Conditions were as described under methods. Values are average ±S.E., of the number of experiments shown in parentheses. Each experiment was performed with different mitochondria preparations. The differences of heat released measured without and with Ca^2+^ in both uncoupled **^(a)^** and coupled **^(b)^** mitochondria were statistically significant (*p*<0.0005). For respiration, **^(c)^** *p*<0.025 and **^(d)^** *p*<0.050. For ATP synthesis, **^(e)^** *p*<0.0025 in uncoupled and non significant in coupled mitochondria **^(f)^**.

BAT mitochondria become coupled when faf-BSA and GDP were included in the assay medium. In these mitochondria the rates of respiration and heat production were decreased, and the rate of ATP synthesis rose to high values (Table 1). Similar to uncoupled mitochondria, Ca^2+^ enhanced the rate of heat production and respiration. Although in coupled mitochondria the rate of heat production was several folds slower than that measured in uncoupled mitochondria, the percent of activation promoted by Ca^2+^ was similar in the two conditions, 60.3% in uncoupled and 54.1% in coupled mitochondria. In coupled mitochondria Ca^2+^ had a discrete effect on the rate of respiration and no effect on the ATP synthesis rate.

### Liver Mitochondria

Different from BAT, liver mitochondria were coupled after the single addition of respiratory substrate (Fig 10. inset), synthesized a considerable amount of ATP but produced only a small amount of heat (Table 2). Different from BAT, Ca^2+^ strongly inhibited ATP synthesis and had no effect on the rate of heat production. Uncoupling of the liver mitochondria with the proton ionophore FCCP abolished the ATP synthesis and promoted a small increase of the heat released (Table 2). These data indicate that the high rate of heat production and the effect of GDP (Figs. 10, 11 and Table 1) are specific features of BAT mitochondria.

**Table 2:** Liver mitochondria.

	EGTA	EGTA+FCCP	Ca^2+^
ATP synthesized µmo/mg. 20 min	1.62±0,19 (10)	<0.10 (3)	<0.10 (10)
Rate of heat released mcal/mg.20 min	51*±5(9)	68*±6(4)	Non detectable (4 experiments)

Conditions were as described under methods. Values are average ±S.E., of the number of experiments shown in parentheses. Each experiment was performed with different mitochondria preparations. When added, the concentration of FCCP was 0.2 µM.

The difference of heat measured in presence of EGTA without and with FCCP were statistically different ^(*)^ *p*<0.050).

### Effect of Different Compounds in BAT Mitochondria

The proton ionophore FCCP (1 µM) dissipated the Δψ formed after the addition of faf-BSA and GDP (Fig. 10), and as a result, enhanced the rate of heat production and inhibited ATP synthesis (Table 3). This was observed in presence and absence of Ca^2+^. On the other hand, oligomycin, a substance that impair the synthesis of ATP by the F1-Fo complex, had practically no effect on the rate of heat production.

**Table 3:** Effect of different drugs on the rates of ATP synthesis and heat production.

Additions	ATP synthesis µmol ATP/mg. 20 min	Heat released mcal/mg. 20 min
None	2.82±0.29 (15)	106±10 (16)
FCCP 1 µM	0.26 (2)	392±30 (4)
Oligomycin 4 µM	0.01 (2)	84±12 (3)
Rotenone 4 µM	0.23 (2)	21±6 (3)
KCN 50 µM	0.08 (2)	14±1 (3)

Experiments were performed with coupled mitochondria with faf-BSA and GDP and in the absence of Ca^2+^. Essentially the same results were obtained in presence of Ca^2+^.

Both in the absence and in the presence of Ca^2+^, an inhibition varying between 70% and 92% of both, ATP synthesis and heat production, were measured after the addition of either 4 µM rotenone or 50 µM KCN, two inhibitors of electron transport chain (complex I and IV, respectively) (Table 3). This indicates that with and without Ca^2+^, the energy for both ATP synthesis and heat production was derived from the electron flux through the cytochrome chain.

### Effect of Lipids in BAT Mitochondria Coupled with faf-BSA and GDP

**Figure 12:**
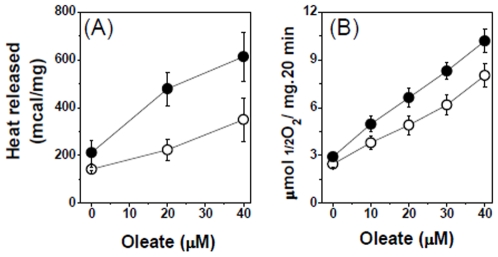
Effect of oleate on (A) the rate of heat production and in (B) oxygen consumption. Experimental conditions were as described in methods. The bars represent S.E. of 5 experiments performed with 5 different BAT mitochondria preparations. Both in A and B, open symbols without Ca^2+^ (1mM EGTA) and closed symbols 2µM Ca^2+^ (0.1 mM EGTA and 0.1 mM CaCl_2_).

In the bibliography it is proposed that lipids antagonize the effect of GDP in BAT UCP1 H^+^ permeability [9], [10], [20]. We now tested the effect of lipids in mitochondria coupled by faf-BSA and GDP, both in the absence and in the presence of Ca^2+^. Oleate, in concentrations up to 40µM did activate the rates of respiration and of heat production. We now show that the activating efect of oleate was more pronounced in presence of Ca^2+^ (Fig 12). In concentration higher than 60µM oleate did impair both respiration and heat production (data not shown) regardless of the presence of Ca^2+^ in the medium.

### Correlation between Oxygen Consumed and Heat Production

In 1979 Ricquier et al. [25], using differential calorimetry, measured the heat produced by uncoupled isolated BAT mitochondria during respiration. These authors found a good correlation between the estimated heat output calculated from oxygen consumption and the heat directly measured with the use of a calorimeter. Based on this finding, during the past 30 years, the direct measurement of heat production using microcalorimetry was scarcely used, the rate of heat production has been calculated from the rate of oxygen consumption and this has been referred to in the bibliography as “indirect calorimetry”.

**Figure 13:**
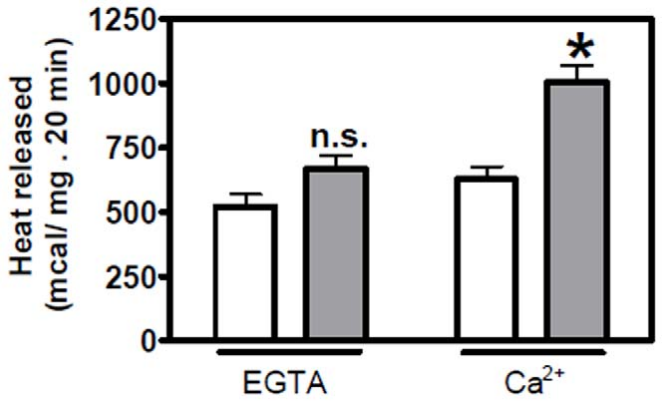
Correlation between heat calculated from the oxygen consumption (white columns) and heat measured (gray column). Heat derived from respiration multiplying the ½ O_2_ consumed by 52.6 kcal/mol, as described in material and methods. Bars are S.E. and *t* test (n.s) *p*<0.10 and (*) *p*<0.0005.

The correlation between respiration and heat measured can be better evaluated in uncoupled mitochondria which practically do not synthesize ATP and the only values to be compared are heat measured with heat calculated from ½ O_2_ consumed. In this report, the aim was to evaluate if the heat measured calorimetrically was solely derived from respiration or, alternatively, if in addition to respiration, other exothermic metabolic routes linked to the cytochrome electron flux could be activated by Ca^2+^. In this case, we should find a discrepancy between “indirect calorimetry” and direct calorimetric measurements (Fig 13). In the absence of Ca^2+^, there was a small difference between the two values but the difference was not statistically significant. This findings is in agreement with Riquier et al [25] early report. In presence of Ca^2+^ however, the amount of heat measured was significantly higher than that estimated from respiration (Fig. 13), indicating that the enhancement of thermogenesis promoted by Ca^2+^ was due to the activation of a different exothermic metabolic route not detected before.

## Discussion

It has been observed in many cell types that the mitochondria are located very close to the cisternae of the endoplasmic reticulum [26], [27]. Continuity of the outer mitochondrial membrane with tubular profiles of endoplasmic reticulum has also been described in different cell types, such as rat hepatocytes, the ciliate *Tetrahymmena pyriformis* [26], in fungi [27], and neural tissue [28]. There is increasing biochemical and morphological evidence demonstrating similarities between the ER and mitochondrial outer membranes, as well as transfer and exchange of materials between the ER and mitochondria [29]. The dynamic interactions of these membranes comprise the phenomena of membrane flow and transformation. It has been proposed that the ER could provide new membranes for mitochondrial growth, and, thus, the role of the ER would be to provide new membrane lipids. Several reports indicated that certain mitochondrial phospholipids were formed in the ER and then transferred to the mitochondrion in liver cells [30]. The localized regions of membrane interaction could raise intermittent bridges, through which cellular macromolecules may be exchanged.

To date, the establishment of a physical connection between the ER and mitochondria in BAT was not previously described. In these cells, the globular ER structures touch the mitochondria. The two structures are apparently pulled together, propitiating the fusion of mitochondrial and ER membranes (Figs. 3, 4, 5). This is different from what was observed in striated muscle where there seems to be no membrane fusion. Small tubular units (tethers) hold the mitochondria and ER together, and communication between the two sub cellular compartments would then be mediated by the tethering structures [3], [4], [6]. A link between BAT and skeletal muscle has been recently reported by Seale et al. [31]. These authors found that the transcriptional regulator PRDM 16 controls a bidirectional differentiation between skeletal myoblasts and brown adipocytes.

The finding of SERCA 1 in BAT mitochondria has led us to study a possible role of Ca^2+^ in BAT mitochondria thermogenesis. It is well established that SERCA 1 uses the energy derived from ATP hydrolysis to simultaneously pump Ca^2+^ across a membrane and to produce heat [32]–[36]. In previous report [16], the effect of Ca^2+^ was studies activating BAT mitochondria with 1 mM ATP. In this report, thermogenesis was activated by respiratory substrate of complex 1 in media without added ATP. At present, it is not clear to us what the role of SERCA 1 in BAT mitochondria is. The following hypothetical possibilities are raised: (a) in the particular case of BAT, Ca^2+^ would be released in mitochondria via the MAM as previously reported for other tissues [4], [6]. However the excess of Ca^2+^ would not be alleviated solely via MAM; it could also be pumped out of the mitochondria by the SERCA 1 located in mitochondrial cristae; (b) SERCA 1 would be involved in the activation of thermogenesis promoted by the addition of low Ca^2+^ concentrations in the assay medium. In favor of this hypothesis are the following findings: (i) the Ca^2+^ concentration needed for half-maximal heat production is in the same range as the Ca^2+^ concentration needed to pump Ca^2+^ in vesicles derived from skeletal muscle sarcoplasmic reticulum [16], [32]; (ii) Ca^2+^ activates only heat production and has no influence in the rate of oligomycin-sensitive ATP synthesis (Table 1); (iii) In the presence of Ca^2+^, there is a significant discrepancy between the rates of oxygen consumption and heat production (Table 1). This could be best seen in uncoupled mitochondria where all energy derived from respiration is dissipated as heat and none is used for oligomycin-sensitive ATP synthesis. Although Ca^2+^ activated both respiration and heat production, the enhancement of respiration was ∼30%, while activation of heat production was ∼60%. The amount of energy derived from each ½ O_2_ consumed is 52.6 kcal [25]. In absence of Ca^2+^, the heat measured was slightly higher than the heat calculated from ½ O_2_ consumed, while in presence of Ca^2+^ it was 60% higher (Fig. 13). This discrepancy may indicate that Ca^2+^ activates a thermogenic process that is not active in the presence of excess EGTA.

The fact that heat production in the presence of either Ca^2+^ or EGTA was impaired by rotenone and cyanide indicates that the activation of heat production by Ca^2+^ is linked to flux of electrons through the cytochrome chain. SERCA 1 has been shown to be able to interconvert different forms of energy to synthesize ATP from ADP and Pi. These include energies derived from a gradient of Ca^2+^, pH, water activity or even thermal energy [33], [37]–[40]. It was also demonstrated that, during ATP hydrolysis, SERCA 1 is able to regulate the flux of energy determining the fraction of energy that is converted into work (Ca^2+^ pumping) and the fraction used for heat production [38]–[40]. Taking in to account that SECA 1 is able to interconvert different forms of energies, the possibility is raised that when activated by Ca^2+^, the mitochondrial SERCA 1 would also be able to absorb part of the energy derived from the electron flux before it reaches oxygen and convert it in to heat. As a result, the rate of heat production would be faster than the rate of ½ O_2_ consumption.

It has been proposed that the Ca^2+^ entering the mitochondria through MAM would activate bioenergetics because Ca^2+^ can activate enzymes in the tricarboxylic cycle, namely α-ketoglutarate and isocitrate dehydrogenase [4]. Acceleration of the tricarboxylic cycle would ultimately lead to an activation of both ATP synthesis and heat production. In favor of this possibility is the finding that in uncoupled mitochondria, a small amount of oligomycin-insensitive ATP was synthesized in the presence of Ca^2+^ (Table 1), and, during the tricarboxylic cycle, one GTP is synthesized from GDP and Pi. The GTP synthesized would then be transformed in to ATP. Against this possibility is the finding that Ca^2+^ activated only the heat production rate and had no effect on the rate of ATP synthesis. If the effect of Ca^2+^ would be derived from activation of the tricarboxylic cycle, then it would be expected that in coupled mitochondria, heat and ATP synthesis would be equally activated.

The proposals discussed above are only working hypotheses, and further experimentation is needed to substantiate these and other possibilities.

## Methods

### Isolation of Mitochondria from Rat BAT and Liver

Wistar rats were treated in accordance with “CEUA - Comissão de Ética em Experimentação Animal – CCS UFRJ”, which follows the guidance of the National Institutes of Health, Bethesda, USA. Our laboratory is certified by the local committee through the project entitled “Interconversão de energia em sistemas biológicos - IBQM 013”. Also experimental animals used in this study are kept in an animal housing facility equally certified by the above committee. Adult male rats were euthanized by decapitation. Brieflly, BAT interscapular and liver were removed and homogenized in a mixture containing 0.32 mM sucrose, 1 mM EDTA, 10 mM MOPS/Tris buffer pH 7.4, and 0.2 mg/ml of non-delipidated BSA (Fraction V-Sigma A7906-50G). The homogenate was centrifuged at 1,330×*g* for 3 min. The supernatant was carefully removed and centrifuged at 21,200×*g* for 10 min. The pellet was re-suspended in the same buffer containing 15% Percoll. A discontinuous density gradient was prepared manually by layering 3-ml fractions of the re-suspended pellet on two preformed layers consisting of 3.5 ml of 23% Percoll above 3.5 ml of 40% Percoll. Tubes were centrifuged for 5 min at 37,700×*g*. The material equilibrating near the interface between 23% and 40% Percoll layer was removed and gently diluted with the isolation buffer described above. After centrifugation at 21,200×*g* for 10 min, the supernatant was decanted, and the pellet was re-suspended in 30 ml buffer containing 0.2 mg/ml non-delipidated BSA and centrifuged at 1,330×*g* for 10 min. The pellet was re-suspended in the isolation buffer using a fine Teflon pestle. During the mitochondria preparation, regular BSA (Fraction V-Sigma) was intentionally used instead of faf-BSA with the aim of obtaining a mitochondrial preparation uncoupled by the lipids that remain attached to the membrane during isolation.

Protein concentration was determined by the Folin-Lowry method using serum albumin as a standard [41].

### Transmission Electron Microscopy and Immunolabeling

BAT was extracted from rats and reduced to three 1-mm pieces, whereas mitochondria from BAT were isolated by differential centrifugation. For routine transmission electron microscopy, samples were fixed in 2.5% glutaraldehyde (v/v) and 5 mM CaCl_2_ in 0.1 M cacodylate buffer (pH 7.2). The pieces were then washed in phosphate buffer saline (PBS) and post-fixed for 60 min in 1% OsO_4_ in cacodylate buffer containing 5 mM CaCl_2_ and 0.8% potassium ferricyanide. After washes in PBS, the material was dehydrated in acetone and embedded in Epon. Ultra-thin (70 nm) sections were stained with uranyl acetate and lead citrate and observed with a JEOL 1210 electron microscope. This procedure allows for high-quality images, but it is not adequate for immunoelectron microscopy because it impairs antibody diffusion through the resin [16].

### Immunoelectron Microscopy

Samples were fixed in 0.7% glutaraldehyde (v/v), 0.1% picric acid, 1% sucrose, 2% paraformaldehyde and 5 mM CaCl_2_ in 0.1 M cacodylate buffer (pH 7.2), dehydrated in ethanol and embedded in Unicryl (Ted Pella, USA). Ultra-thin sections were collected in nickel grids with 300 mesh and quenched in 50 mM NH_4_Cl for 30 min. Afterwards, the samples were incubated in the presence of monoclonal anti-Serca-1 antibody (clone IIH11, Affinity BioReagents, Inc., Brazil). After several washes in PBS-1% albumin, sections were incubated in the presence of 10-nm gold-labeled goat anti-mouse IgG (BB International, UK), washed, and observed with a JEOL 1210 electron microscope. This method allows for an adequate diffusion of the antibody but it decreases the preservation of the material due to light fixation, and, therefore, it decreases the quality of the image [16].

### ATP Synthesis

ATP synthesis was determined measuring the incorporation of ^32^P_i_ into [γ-32P] ATP, with the excess of ^32^P_i_ being extracted from the medium as phosphomolybdate with 2-butanol benzene [42]. In order to ensure that the ATP synthesized was derived from ^+^H gradient and ATP synthase, synthesis was measured in the presence and absence of oligomycin (0.5 up to 1 µM). In control experiments, we measured ATP synthesis simultaneously using ^32^P_i_ and using hexokinase and glucose-6 phosphate dehydrogenase [38], [42]. In these control experiments, samples were cooled, mitochondria were removed by centrifugation, and the amount of ATP in the media was measured using the two methods. The values of ATP found were the same regardless of the method used.

*Heat of reaction*. This was measured using an OMEGA Isothermal Titration Calorimeter from Microcal, Inc. (Northampton, MA). The calorimeter sample cell (1.5 ml) was filled with reaction medium, and the reference cell was filled with Milli-Q water. After equilibration at 35°C, the reaction was started by injecting mitochondria into the sample cell, and the heat change was recorded for 20 min. The volume of mitochondria suspension injected in the sample cell varied between 15 and 45 µl, and the mitochondrial protein concentration in the calorimeter cell varied between 10 and 30 µg/ml. The heat change measured during the initial 3 min after mitochondria injection was discarded in order to avoid artifacts such as heat derived from the dilution of the mitochondria suspension in the reaction medium and binding of ions to mitochondria [18], [34]. Negative heat values indicate that the reaction is exothermic, and positive values indicate that it is endothermic. The microcalorimeter can also be used for binding measurements. In this case the gases diluted in the test solution are usually removed in a vacuum before use. This step was not done in our measurements, and less than 30% of the oxygen available in solution was used during the experimental measurements.

### Oxygen Uptake Measurements

Oxygen consumption rates were measured using a high-resolution respirometry (OROBOROS Oxygraph-O2K). The Oxygraph-2k is a two-chamber titration-injection respirometer with a limit of oxygen flux detection of 1 pmol/sec · ml. The electrode was calibrated between 0 and 100% saturation with atmospheric oxygen at 37°C. The BAT mitochondrial concentration used varied between 20 and 50 µg/ml.

### ΔΨ Determination

Mitochondrial membrane potential was measured using the fluorescence signal of the cationic dye safranine O (10 µM), which is accumulated and quenched inside energized mitochondria. For the fluorescence measurement, the mixture contained in a 2-ml cuvette was excited at 495 nm, and the emission was read at 586 nm.

### Calculation of Heat Derived from Oxygen Consumption

This was done as previously described, multiplying the ½ O_2_ consumed by 52.6 kcal/mol [25].

### Gel Electrophoresis and Western Blot

Protein samples were resolved on polyacrylamide gels: 13% for UCP 1 proteins, according to Laemmli [43]. The immunoblots were revealed using an ECL PLUS detection kit from Amersham-Pharmacia Biotech, UK. Polyclonal anti-UCP1 antibody (ab10983) was obtained from Abcam (USA).

### Experimental Conditions

All experiments were performed at 35°C, pH 7.4. All solutions used contained 20 mM HEPES or 50 mM MOPS/Tris buffer pH 7.4, 0.2 mM ADP, 2 mM Pi, and 4 mM MgCl_2_. The respiratory substrate used was 1 mM pyruvate plus 1 mM malate. When indicated, 1 mM GDP or 1 mg/ml faf-BSA were included in the medium. For experiments in the absence of Ca^2+^, 1 mM EGTA was included in the media. For experiments in the presence of Ca^2+^, a mixture of 0.1 mM EGTA and 0.1 mM CaCl_2_ was used, which yielded a free Ca^2+^ concentration of 2 µM. This concentration was calculated as described previously [44].
